# Supplementary material for: Label-Free Direct Detection of Saxitoxin Based on a Localized Surface Plasmon Resonance Aptasensor
Source: Toxins (Basel). 2019 May 15;11(5):274. doi: 10.3390/toxins11050274 (PMC6563244; doi:10.3390/toxins11050274)
Supplement: Supplementary file 1 [file toxins-11-00274-s001.pdf]

Supplementary Materials: Label-free direct detection of saxitoxin based on a localized surface plasmon resonance aptasensor

Su-Ji Ha, Jin-Ho Park, Bobin Lee and Min-Gon Kim

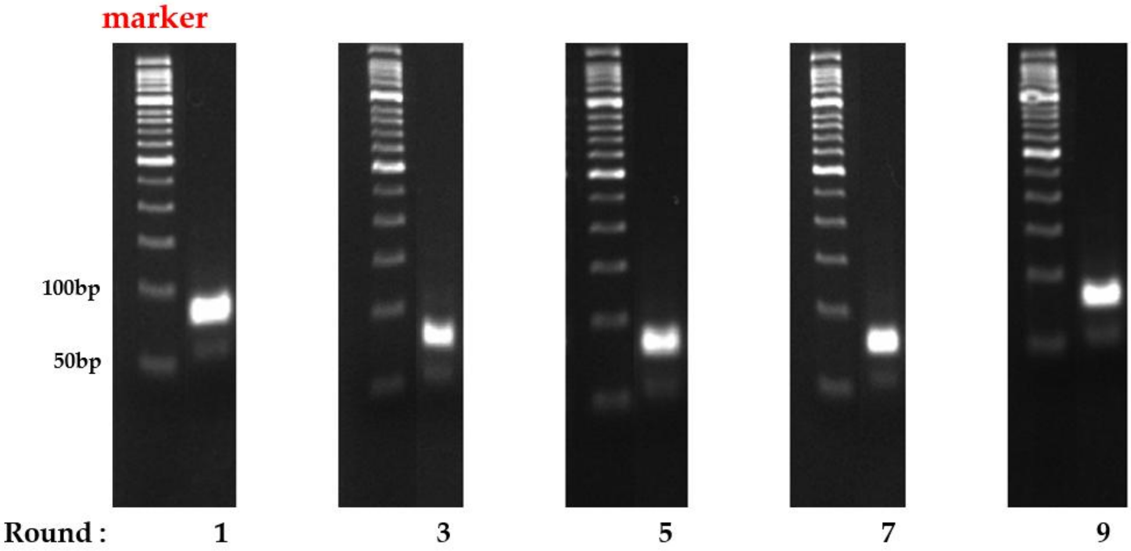

Figure S1. Agarose gel images of the GO-SELEX rounds (76mer = random sequence library size).

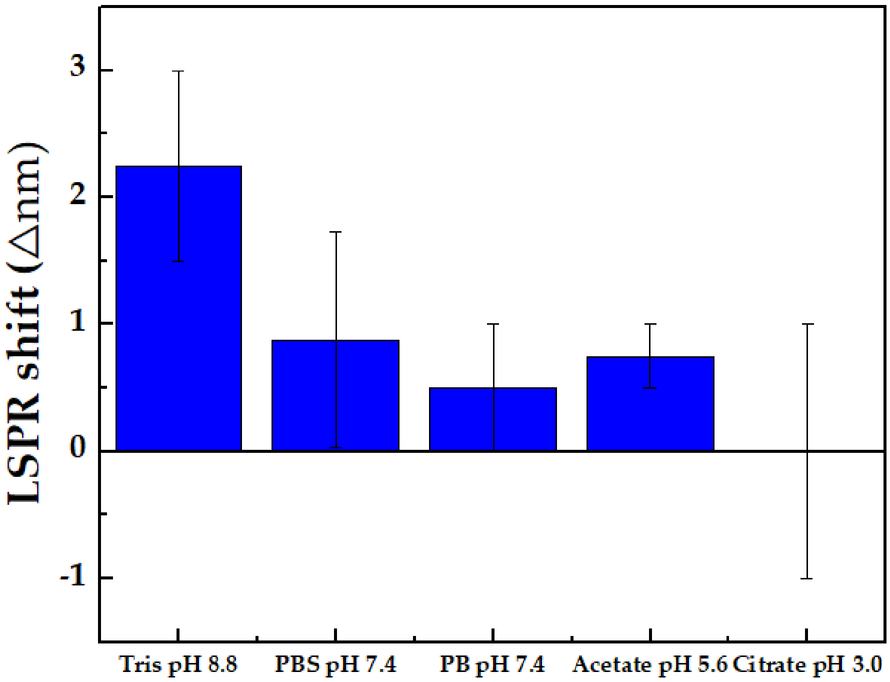

Figure S2. Comparison of non-specific absorption to LSPR chip fixed with only poly T_3 spacers (n = 3).

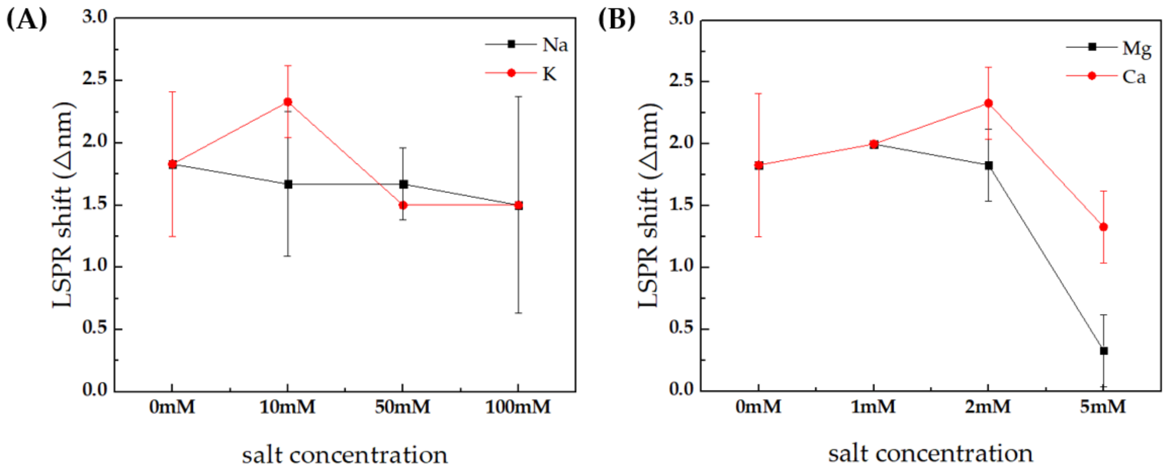

Figure S3. Comparison of the effect of salts used in the working buffer. (**A**) The LSPR shift of the monovalent cations. (**B**) The LSPR shift of the divalent cations. (n = 3).

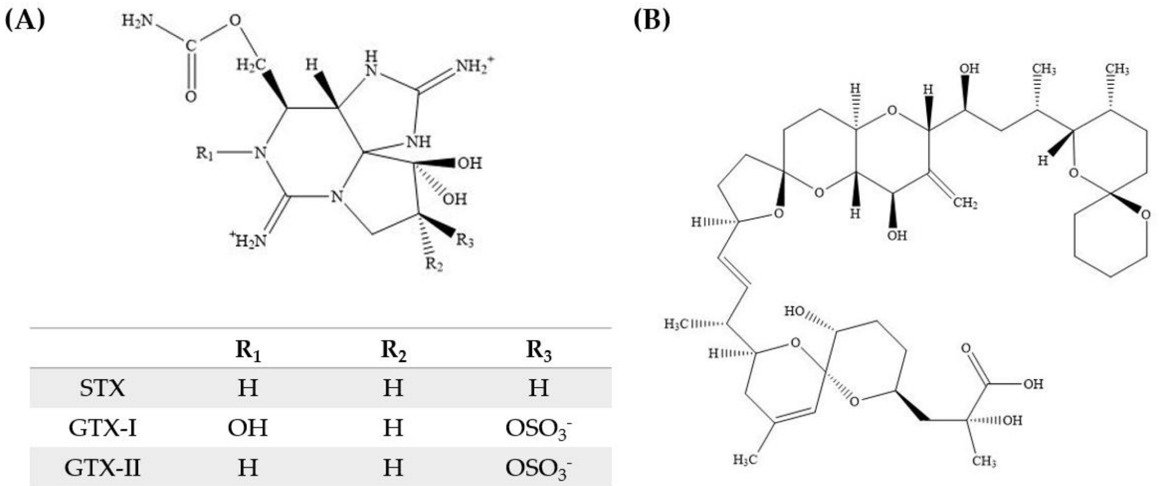

	R_1	R_2	R_3
STX	H	H	H
GTX-I	OH	H	OSO_3^-
GTX-II	H	H	OSO_3^-
GTX-III	H	OSO_3^-	H
GTX-IV	OH	OSO_3^-	H
NEO	OH	H	H

Figure S4. Chemical structure of STX analogs. (**A**) GTX complex and neoSTX. (**B**) Okadaic acid.

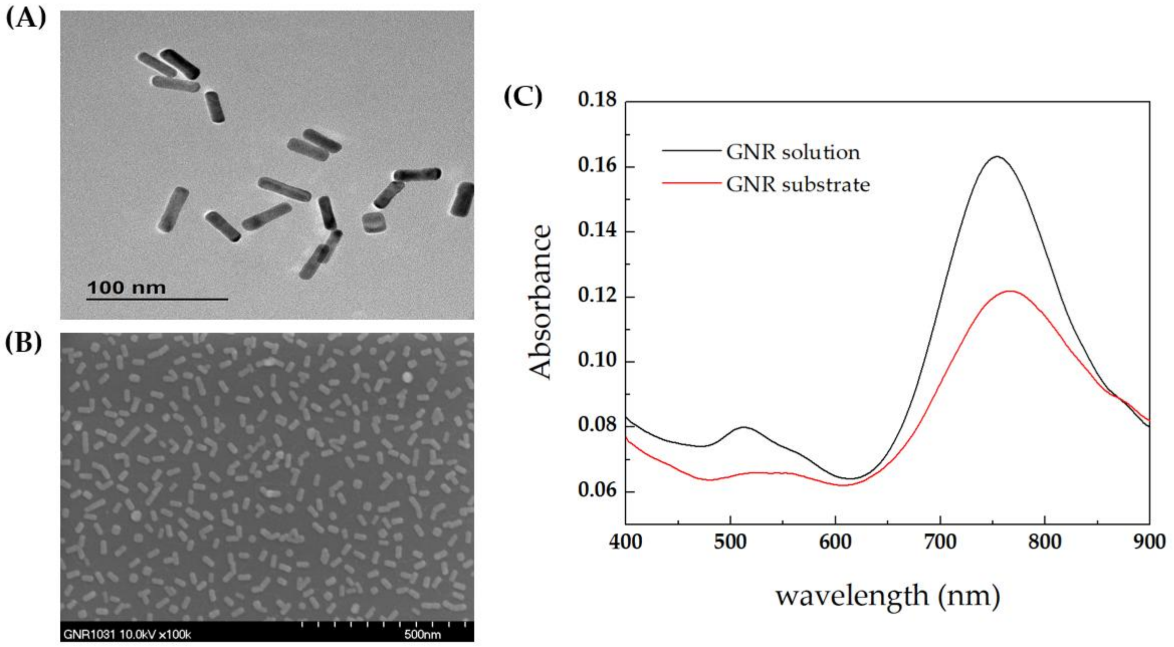

Figure S5. (**A**) TEM image of the synthesized GNR solution. (**B**) SEM image of the GNR chip. (**C**) Absorption spectra of GNR solution (black) and GNR immobilized on the chip (red).
